# Predictors of Effectiveness and Adherence in a Multimodal Obesity Treatment Program for Children and Adolescents in Routine Care

**DOI:** 10.3390/nu15010136

**Published:** 2022-12-28

**Authors:** Julius Lars Breinker, Anika Kaspar, Elena Sergeyev, Antje Körner, Wieland Kiess, Anja Hilbert

**Affiliations:** 1 Integrated Research and Treatment Center Adiposity Diseases, Behavioral Medicine Research Unit, Department of Psychosomatic Medicine and Psychotherapy, University of Leipzig Medical Center, 04103 Leipzig, Germany; 2 Center for Pediatric Research, Department of Pediatrics, University of Leipzig Medical Center, 04103 Leipzig, Germany; 3 LIFE Research Center for Civilization Diseases, University of Leipzig Medical Center, 04103 Leipzig, Germany

**Keywords:** obesity, children, adolescents, multimodal, treatment, predictors, efficacy, adherence, COVID-19, routine care

## Abstract

Multimodal obesity treatments for children and adolescents generally showed only small to modest treatment effects and high dropout rates. Potential variations by patients’ clinical and sociodemographic factors remain, however, largely unclear. For this reason, our study analyzed psychological, physical, and sociodemographic predictors of treatment success and adherence in a multimodal obesity treatment over 12 months. The intent-to-treat sample included *n* = 361 children and adolescents (ages 3–17 years), of which *n* = 214 or 59.28% of patients completed treatment. A younger age and, in the sensitivity analysis, additionally a greater eating disorder psychopathology and treatment initiation before COVID-19 pandemic predicted greater BMI-SDS reductions (Body Mass Index-Standard Deviation Score). In contrast, predictors of treatment adherence were not found. The results underline the importance of early treatment of juvenile obesity. Additionally, eating disorder psychopathology includes restrained eating, which implies the ability to self-regulate eating behavior and therefore may have a positive effect on the treatment goal of controlled food intake. Challenges from altered treatment procedures due to the COVID-19 pandemic nonetheless remain.

## 1. Introduction

Overweight and obesity have a high prevalence of 9.5% and 5.9%, respectively, in the 3- to 17-year-old age group in Germany [1] and pose a significant challenge to patients and the healthcare system. Overweight and obesity in children and adolescents are defined by Body Mass Index (BMI, kg/m^2^) percentiles of >90 or >97%, respectively [2], based on a German reference population of boys and girls aged 0 to 18 years [3]. Since the beginning of the COVID-19 pandemic, the weight status of children and adolescents has deteriorated [4], with the percentage of children and adolescents classified as overweight or obese increasing [5] and preexisting juvenile obesity aggravated further [4]. Multimodal lifestyle interventions with behavioral and family interventions, focusing on diet and exercise, are most commonly recommended [2]. However, international systematic reviews have shown that randomized controlled studies on multimodal obesity treatments for children and adolescents are only moderately efficacious [6,7]. Additionally, in the outpatient routine care context, multimodal obesity treatments for children and adolescents face challenges, resulting in reduced weight loss effects [8], uncertain long-term treatment effects [6,7], and high dropout rates [9].

Knowledge of factors that can predict treatment success and adherence at treatment initiation is required in order to enable long-term therapeutic success, make obesity treatment as beneficial as possible for patients and parents, and keep it cost-efficient for the healthcare system. Previous studies primarily focused on psychological, physical, family, and sociodemographic factors. However, since many of the investigated parameters can hardly be modified or addressed by the affected children and adolescents [9,10,11], it is recommended that modifiable influencing factors for success and adherence in the context of juvenile obesity treatment be deemed the focus [9,10]. While parental influence is stated as being central to treatment initiation, the importance of children or adolescents in relation to their own adherence to treatment was highlighted [10].

The available evidence regarding influencing factors affecting treatment success and adherence in children and adolescents with obesity is very heterogeneous. At baseline before treatment, psychological predictors associated with greater BMI(-SDS) reduction were low emotional problems [12] and low hyperactivity/attention deficits [12], among others. In contrast, no association was found with self-concept [13] and general quality of life [14]. In this context, the Body Mass Index-Standard Deviation Score (BMI-SDS) describes the individual deviations from the mean values for age and sex. As far as we could determine, the influence of eating disorders, i.e., binge-eating symptoms, has been studied exclusively in adults, with the evidence mostly supporting a negative influence on long-term weight loss success [15,16,17]. In previous studies, motivation produced mixed results as a predictor for weight loss success, often with only parents being surveyed [9]. However, the need for further research into treatment motivation in children and adolescents with obesity was stressed [11]. The results for child and adolescent age and sex as predictors for successful weight loss have been contradictory [13,18,19,20]; one study found significantly greater treatment success in adolescent girls compared with treatment success in children and boys [21]. As an anthropometric parameter, a higher baseline weight status did not predict a greater BMI(-SDS) reduction within the scope of juvenile obesity therapies [14,19,20], despite a conflicting nature of the evidence [18]. Finally, the COVID-19 pandemic was highlighted as a negative influence on weight loss success in the context of juvenile obesity treatment [22].

Psychological predictors associated with low treatment adherence in terms of premature dropout from treatment included reduced behavioral competencies [11], greater conduct problems [23] and social anxiety [24], increased internalizing behavior [9], and decreased body satisfaction [23]. In contrast, self-perception of one’s own competencies [13], externalizing behavior [9], and health-related quality of life [23] had no predictive value. The findings related to psychosocial stress factors as predictors for adherence in juvenile obesity treatment are conflicting [9,18]. Although bulimic symptoms were shown to be a negative predictor for adherence to juvenile obesity treatments [24], to the best of our knowledge, the impact of binge-eating symptoms on treatment adherence has only been studied in adult obesity treatments and has shown mixed results [25]. While there is mixed evidence for the age of juvenile patients as a predictor [9,11,18,23], sex did not predict treatment adherence [9,11,13,23], but one study showed an interaction with more frequent dropouts in young male patients in comparison with that of adolescents or girls [21]. Furthermore, some studies identified a higher baseline weight as a negative predictor for treatment adherence [13,23]; however, the evidence is again conflicting [9,10,11].

For future analysis of juvenile obesity treatment adherence, Spence et al. primarily called for more detailed descriptive statistics reporting by subgroups, a uniform definition of treatment adherence, and a content-based selection of variables for analysis [26]. Furthermore, limited evidence outside the United States or from countries with universal health insurance was pointed out [9,10,26], as studies from the United States or countries with comparable health insurance systems repeatedly showed private versus public health insurance to be a positive predictor of treatment adherence and, in some cases, treatment success [9,10,21]. Overall, previous studies indicated contradictory results regarding predictors of success and adherence in the context of juvenile obesity treatment, highlighting the need for further evidence. Consequently, the aim of this study was to identify the baseline predictors of treatment success and adherence for a multimodal juvenile obesity treatment in the outpatient routine care setting using standardized and validated assessments. The assessed parameters included modifiable and therapeutically addressable general and obesity-related psychological factors, as well as the physical and sociodemographic parameters of the patients. Furthermore, the influence of treatment initiation on treatment success and adherence before versus during the COVID-19 pandemic was investigated.

Against the backdrop of these research findings, this study assumed that low overall psychopathology, high physical and mental quality of life, low general physical complaints, low eating disorder psychopathology, less binge eating, high treatment-related motivation, treatment initiation before the COVID-19 pandemic, lower baseline BMI-SDS, higher age, and female sex are significant positive predictors for treatment success and adherence.

## 2. Materials and Methods

### 2.1. Participants and Ethics

The sample was comprised of children and adolescents participating in the “Leipziger Adipositasmanagement” obesity treatment program, with treatment initiation between June 2015 and December 2020. The participants or their parents contacted the Integrated Research and Treatment Center Adiposity Diseases (IFB) at the Department of Pediatrics at the University of Leipzig Medical Center, Leipzig, Germany for weight loss treatment. People aged ≤18 years and with a BMI-SDS percentile of ≥90 or a previous BMI-SDS increase of ≥1 per year were included in the treatment program. The children, adolescents, and parents confirmed their consent to the use of their data within the scope of studies via written informed consent or assent forms. The study was conducted according to the guidelines of the Declaration of Helsinki and approved by the Ethics Committee of the University of Leipzig.

### 2.2. Multimodal Obesity Treatment

The “Leipziger Adipositasmanagement” obesity treatment program was funded and implemented in the routine care setting by University of Leipzig Medical Center in cooperation with the regional health insurance provider for Saxony and Thuringia, AOK PLUS (Allgemeine Ortskrankenkasse Sachsen und Thüringen), within the framework of the integrated healthcare provision in accordance with § 140 SGB V [27,28]. After comprehensive anamnesis, the individual treatment program was derived in accordance with the S3 guideline for Obesity in Childhood and Adolescence by the German Guidelines Workgroup (AGA) [2]. The program included the following areas: medicine, psychology (individual sessions if indicated or requested by the family), nutrition (8 group sessions over 16 weeks and 6 individual sessions over 32 weeks), exercise (personal therapy plan, weekly sessions over 48 weeks), and social work (according to personal needs). The treatment program covered approximately 12 months, and a transition to an aftercare phase over approximately 3 years was possible.

During the regional and national lockdowns, the effects of the COVID-19 pandemic manifested as appointment cancellations and an adjustment of therapy procedures and modalities. Structured therapeutic procedures resumed at the end of the lockdowns.

### 2.3. Data Collection

The height and weight of the patients were objectively measured and used to calculate BMI-SDS and the weight loss success from treatment initiation (t_0_) to end (t_1_) (Δ-BMI-SDS) after 12 months. The variable treatment initiation during the COVID-19 pandemic was dichotomously defined as treatment beginning before versus after 1 March 2020. Treatment adherence was determined dichotomously as dropout versus completer, whereby dropout was defined as treatment discontinuation between t_0_ and t_1_ and completer as achieving t_1_ with the conclusion of the treatment program. Psychological questionnaires were completed as self-reported for adolescent patients aged 12 years and older, and as parent-reported for children up to and including 11 years of age.

Psychopathology was assessed using the Strengths and Difficulties Questionnaire (SDQ) [29]. The questionnaire was comprised of 5 subscales to survey emotional symptoms, conduct problems, hyperactivity/inattention, peer relationship problems, and prosocial behavior, using 25 items with responses given using a 3-point rating scale (0 = “Not true” to 2 = “Certainly true”). The total difficulties score was calculated using the 4 psychopathological subscales (without prosocial behavior), with higher values indicating greater overall psychopathology (Cronbach’s α = 0.82; McDonald’s ω = 0.81; here and hereinafter referring to the presented sample).

Physical and mental quality of life were surveyed using the respective subscales of the German questionnaire Health-Related Quality of Life in Children and Adolescents (KINDL-R) [30], each of which is comprised of 4 items on a 5-point rating scale (1 = “Never” to 5 = “All the time”) with reference to the previous week. Value transformations produced scale values between 0 and 100, with higher values indicating a better quality of life (KINDL-R Physical: Cronbach’s α = 0.69; McDonald’s ω = 0.70; KINDL-R Mental: Cronbach’s α = 0.68; McDonald’s ω = 0.69).

General physical complaints were measured using the Health Behavior in School Children–Symptom Checklist (HBSC-SCL) [31]. Using a 5-point scale (0 = “About every day” to 4 = “Rarely or never”), 8 items were used to record the general symptom load, including headaches, abdominal pain, and back pain, with reference to the previous 6 months. A lower total value indicated more symptoms (Cronbach’s α = 0.79; McDonald’s ω = 0.79).

The specific eating disorder psychopathology was measured using the Child Eating Disorder Examination-Questionnaire8 (ChEDE-Q8) [32]. Referencing the previous 28 days, 8 items were answered using a 7-point scale (0 = “Characteristic was not present” to 6 = “Characteristic was present every day or in extreme form”). Each of the following subscales was recorded using 2 items: restrained eating, eating concern, shape concern, and weight concern. A higher overall mean value indicated a greater general eating disorder psychopathology (Cronbach’s α = 0.84; McDonald’s ω = 0.85).

The frequency of binge-eating episodes was assessed using the corresponding item in the Child Eating Disorder Examination-Questionnaire (ChEDE-Q) [32]. The number of binge-eating episodes, referred to as the consumption of an objectively large amount of food and a subjective feeling of loss of control, within the last 28 days was surveyed.

Treatment motivation of the patient was assessed using 3 items: motivation, willingness, and confidence for short- and long-term health-promoting behavior change, using an 11-point scale (0 = “Not at all” to 10 = “Very much”). Principal component analysis supported a one-factorial structure of treatment motivation, and therefore the mean value of the items was used. A higher mean value indicated greater treatment motivation.

### 2.4. Data Analysis

The influence of predictor variables on treatment success, measured as Δ-BMI-SDS from t_0_ to t_1,_ was analyzed using hierarchical multiple linear regression analysis. The predictors for the first model encompassed age, sex, AgeXSex interaction, treatment initiation during the COVID-19 pandemic, and t_0_-BMI-SDS. The second model additionally encompassed the psychological predictors. The predictor analysis on treatment adherence (dropout/completer) was carried out using hierarchical logistic regression analysis and models similar to those in the multiple linear regression analysis.

In the multiple linear regression analysis, missing values for the variable t_1_-BMI-SDS of the dropout group were replaced by multiple imputation [26] as part of an intent-to-treat approach. Imputed values were calculated using the SPSS multiple imputation procedure (50 imputations), adjusting for patient age, sex, and t_0_-BMI-SDS. For the sensitivity analysis, the regression analysis was repeated using complete datasets only (completer analysis) and additionally with use of t_0_-BMI-SDS values for missing t_1_ values (baseline observation carried forward, BOCF). Due to different scaling of the continuous predictors, the variables were mean-centered before inclusion in the regression analyses.

As effect sizes, for logistic regression, the odds ratio (*OR*) was reported; and *d* for *t*-tests, φ for chi-squared tests, and *R*^2^ for multiple regression were reported and interpreted as follows [33]: small: *d* ≥ 0.20, φ ≥ 0.10, and *R*^2^ ≥ 0.02; moderate: *d* ≥ 0.50, φ ≥ 0.30, and *R*^2^ ≥ 0.13; large: *d* ≥ 0.80, φ ≥ 0.50, and *R*^2^ ≥ 0.26. A power analysis indicated a power of 1 − β > 0.99 for the multiple linear regression analysis and a minimum sample size of *n* = 340 [34] for the logistic regression analysis to identify main effects of medium-effect size. The multiple linear regression analysis with the completer sample (*n* = 214) showed a power of 1 − β = 0.98.

The statistical analysis was conducted using IBM SPSS Statistics Subscription Version 28.0.1.1 for Windows, and a two-tailed significance level of 0.05 was applied to all statistical tests.

## 3. Results

### 3.1. Participants

A total of *n* = 363 children began obesity treatment at t_0_, of which *n* = 2 were excluded for outliers in predictor and/or outcome variables. The intent-to-treat sample included *n* = 361 children, of which *n* = 214 or 59.28% of patients completed treatment (completer analysis), while *n* = 147 patients discontinued treatment prematurely (dropout). Reasons for dropout included lack of time, lack of motivation from patients or parents, other personal reasons on the part of the patients, and inability to contact the families or missing several appointments on the part of the clinic. In the completer sample, *n* = 124 patients displayed a BMI-SDS reduction, with Δ-BMI-SDS values between −0.01 and −1.14, while *n* = 90 patients displayed an unchanged or increased weight, with a Δ-BMI-SDS value between 0.00 and 0.79. Treatment success according to evidence-based guidelines (Δ-BMI-SDS ≤ −0.20) [2] was achieved by *n* = 63 completers.

At t_0_, the participants were 10.68 years old (*SD* = 3.19), and 53.19% were female (t_1_: age: *M* = 11.74; *SD* = 3.24; 53.27% female; see Table 1). There was no difference between the sexes in terms of age and t_0_-BMI-SDS: *t*(359) = 0.78; *p* = 0.43; *d* = 0.08 and *t*(359) = 0.12; *p* = 0.91; *d* = 0.01. There was no difference between dropouts and completers with regard to age or sex: *t*(359) = 0.68; *p* = 0.50; *d* = 0.07 and χ^2^(1; *n* = 361) < 0.01; *p* = 0.97; φ = 0.00. There was also no difference in t_0_-BMI-SDS between dropouts and completers: *t*(359) = −0.05; *p* = 0.96; *d* = −0.01.

### 3.2. Treatment Success

The results of the multiple linear regression analysis for weight loss prediction in the intent-to-treat sample showed a significant influence of first-model predictors on treatment success (Δ-BMI-SDS), with a small to moderate explanation of variance (see Table 2). In this model, a lower age significantly predicted greater treatment success. In the second model, the linear regression analysis was significant, with an increased effect size but a small to moderate explanation of variance. Again, a lower age predicted significantly greater weight loss.

In the sensitivity analysis based on the completer sample, in the first model, a lower age and treatment initiation before the COVID-19 pandemic predicted greater treatment success, with a small to moderate explanation of variance. In the second model, along with a moderate to large explanation of variance, additionally a greater eating disorder psychopathology predicted greater treatment success. In the sensitivity analysis using the BOCF sample, in the first model, lower age and treatment initiation before the COVID-19 pandemic predicted greater weight loss success. In the second model, a greater eating disorder psychopathology additionally predicted greater treatment success. Both models displayed a small to moderate explanation of variance.

An exploratory post-hoc ChEDE-Q8 subscale analysis showed that more restrained eating and shape concern were the factors most positively associated with weight loss success (see Table 3). However, the effects were very small.

### 3.3. Treatment Adherence

The only significant predictor of treatment adherence was shown to be treatment initiation before the COVID-19 pandemic. However, the logistic regression analysis (see Table 4) showed no significant predictive value overall in the first or second model: χ^2^(5; *n* = 361) = 8.28; *p* = 0.14; Nagelkerke *R*^2^ = 0.04 and χ^2^(12; *n* = 361) = 10.80; *p* = 0.55; Nagelkerke *R*^2^ = 0.05.

## 4. Discussion

This study analyzed predictors for success and adherence in a multimodal obesity treatment for children and adolescents. A larger BMI-SDS reduction in the intent-to-treat sample was observed in younger patients. In the sensitivity analysis, a greater treatment success was predicted by a younger age, treatment initiation before the COVID-19 pandemic, and greater eating disorder psychopathology. For treatment adherence, the predictor models were found to be non-significant, although treatment initiation before the COVID-19 pandemic significantly predicted treatment adherence.

**Figure 1:**
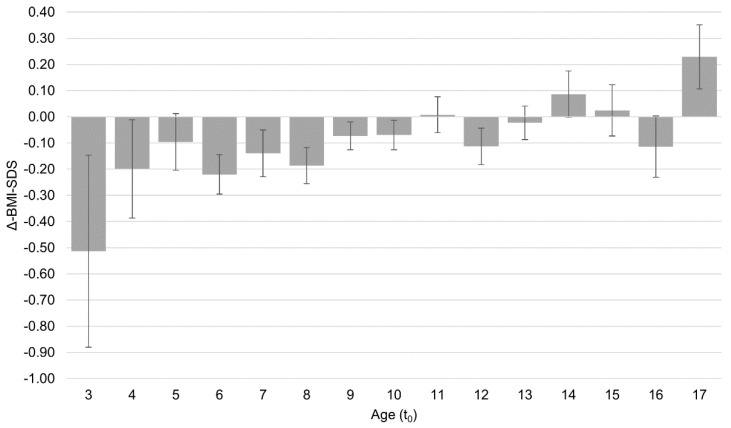
Patient age at treatment initiation (t_0_) and BMI-SDS reduction (*M* ± *S.E.*) at end of treatment (t_1_) (Intent-to-treat sample with multiple imputation, *n* = 361). BMI-SDS = Body Mass Index-Standard Deviation Score; M = mean; S.E. = standard error of mean.

The greater treatment success of younger patients (see Figure 1) supplements the conflicting data from previous studies [18,19,20]. Despite age not being a directly modifiable factor [9,10], the findings underscore the great significance of the early initiation of treatment. The lower treatment success displayed by older patients in our sample may stem from longer exposure to obesity-related symptoms and higher baseline psychopathology in adolescents compared with those in children with obesity [35]. The negative influence of the COVID-19 pandemic on treatment success shown in the sensitivity analysis confirmed the findings of a recent study [22] and may stem from therapy setting changes, especially during the lockdowns (March–May 2020, November 2020–May 2021, and end of 2021). Factors such as missing appointments, changes to treatment modalities, etc., may have limited treatment intensity and quality. Additionally, when the COVID-19 pandemic began, children and adolescents were confronted with not only major psychosocial stresses but also the loss of exercise opportunities in school and in their free time.

To the best of our knowledge, the positive influence of eating disorder psychopathology on treatment success in the sensitivity analysis was found for the first time for children and adolescents. The results of the post-hoc ChEDE-Q8 subscale analysis contrast with the results of adult obesity treatment, where shape concern had a negative impact on long-term weight loss success and adherence [17,36]. Restrained eating may be beneficial for the treatment aim of controlled food intake and may facilitate weight loss success in children and adolescents, although it was not associated with therapy success or adherence in adults [17,36].

According to our knowledge, a lack of influence of the physical symptom load and motivation on the success of juvenile obesity treatment has been demonstrated for the first time. Regarding motivation, this could be due to the importance of parental motivation and may represent a difference to the motivational structure in adult obesity therapies [37]. The lack of influence of physical complaints could indicate a low burden of obesity-related symptoms in the children and adolescents studied, especially considering the general psychopathology and psychological quality of life, which were also not predictive, and the young mean age of 10.68 years at t_0_ in our sample. Regarding general psychopathology and quality of life, this contradicts previous evidence [12,13,14], although these studies predominantly examined adolescent patients. However, physical quality of life tended to have a negative, barely non-significant impact on treatment outcome in our study.

The lack of significance of the logistic regression analysis in identifying predictors of treatment adherence adds to the conflicting evidence regarding age, sex, baseline weight status, psychosocial stress factors, and externalizing behavior [9,10,13,18,21,23]. The findings of earlier studies on the predictive value of eating disorder psychopathology [24], behavioral competencies [11], general and subjective health [9,23], and internalizing behavior [9] for treatment adherence were not replicated either in this study. Nevertheless, this study demonstrated a lack of influence of binge eating and treatment motivation on treatment adherence in children and adolescents for the first time. The greatest negative influence on treatment adherence was treatment initiation during the COVID-19 pandemic, with an OR of 0.39, despite a lack of significance of the overall model.

The results must be interpreted by taking into account study limitations. As a result of the dichotomization, the predictor variable treatment initiation during the COVID-19 pandemic captured the impact of the COVID-19 pandemic only to a limited extent. Therefore, more detailed analyses should be performed, with comparisons before versus after pandemic onset, which could not be performed in our study because of small sample sizes since pandemic onset (*n* = 52). Additionally, the treatment completion decreased from 62.14% of patients whose treatment began before onset of the COVID-19 pandemic to 42.31% of those who began after the onset of the pandemic. The study sample size also implied that the regression analyses could not take into account an interaction of the psychological predictors with patient age and sex. Nevertheless, the power analysis confirmed a very high power for the regression analyses in this study. Since psychological questionnaires were only performed by self-reporting or parent-reporting, depending on patient age, this may limit the comparability of the psychological data. Finally, the scales for physical and mental quality of life generally tended to show low reliability values related to a low number of items per scale (4 items).

The strengths of this study include the application of validated psychometric scales and the reporting of comprehensive descriptive statistics by subgroups. This also allows for comparisons with other study populations and outcomes [26]. The broad range of psychological constructs and the analysis on treatment success and adherence in a routine care sample supplements earlier studies that often only analyzed single scales in different populations with different treatment regimens. The wide age range of weight loss patients enabled analysis from childhood through adolescence.

Future studies should examine treatment success and adherence based on age groups and sex. Additionally, analyses from before and after the beginning of the pandemic should be viewed separately, and comparative studies carried out.

The results of our study regarding the predictors of weight loss success and treatment adherence underline the importance of the early treatment of juvenile obesity. Furthermore, eating disorder psychopathology includes restrained eating, which implies the ability to self-regulate eating behavior and therefore may have a positive effect on the treatment goal of controlled food intake. Challenges from the altered treatment procedures due to the COVID-19 pandemic nonetheless remain.

## Figures and Tables

**Table 1:** Descriptive statistics for the intent-to-treat sample and the completer/dropout subgroups.

	Intent-to-Treat (*n* = 361) *M* (*SD*)	Completer (*n* = 214) *M* (*SD*)	Dropouts (*n* = 147) *M* (*SD*)
Age	10.68 (3.19)	10.58 (3.30)	10.82 (3.03)
Female, %	53.19	53.27	53.06
t_0_-BMI-SDS	2.50 (0.55)	2.50 (0.57)	2.50 (0.52)
Δ-BMI-SDS	−0.07 (0.34)	−0.08 (0.34)	-
SDQ Total	13.01 (6.19)	13.00 (6.07)	13.01 (6.38)
KINDL-R Physical	70.54 (18.71)	70.40 (18.87)	70.76 (18.53)
KINDL-R Mental	75.60 (17.42)	75.67 (17.51)	75.49 (17.36)
HBSC-SCL	24.79 (6.01)	25.07 (5.85)	24.40 (6.23)
ChEDE-Q8	1.94 (1.39)	1.86 (1.38)	2.06 (1.40)
Binge Eating	1.51 (3.85)	1.56 (4.01)	1.43 (3.61)
Motivation	6.83 (2.15)	6.77 (2.01)	6.92 (2.34)

Notes: Intent-to-treat sample with multiple imputation; BMI-SDS = Body Mass Index-Standard Deviation Score; SDQ = Strengths and Difficulties Questionnaire; KINDL-R = Health-Related Quality of Life in Children and Adolescents; HBSC-SCL = Health Behavior in School Children–Symptom Checklist; ChEDE-Q8 = Child Eating Disorder Examination-Questionnaire8; Binge Eating = average frequency of binge-eating episodes in the last 28 days.

**Table 2:** Linear regression analysis for predictors of Δ-BMI-SDS (unstandardized coefficients).

	Intent-to-Treat (*n* = 361)					Completer (*n* = 214)					BOCF (*n* = 361)				
	*F*	*B*	*SE B*	*p*	*R* ^2^	*F*	*B*	*SE B*	*p*	*R* ^2^	*F*	*B*	*SE B*	*p*	*R* ^2^
Model 1	2.34			<0.01	0.08	4.16			<0.01	0.11	4.30			<0.01	0.07
Constant		−0.09	0.04	0.01			−0.11	0.03	<0.01			−0.07	0.02	<0.01	
Age		0.03	0.01	<0.01			0.03	0.01	<0.01			0.02	0.01	<0.01	
Sex		0.03	0.05	0.60			0.04	0.05	0.37			0.02	0.03	0.40	
AgeXSex		−0.01	0.01	0.32			−0.03	0.02	0.10			−0.02	0.01	0.10	
t_0_-BMI-SDS		0.00	0.04	0.96			−0.01	0.04	0.87			0.00	0.03	0.92	
COVID-19 pandemic		0.09	0.07	0.20			0.23	0.09	0.01			0.11	0.04	0.01	
Model 2	2.94			<0.01	0.11	3.00			<0.01	0.18	3.00			<0.01	0.11
Constant		−0.09	0.04	0.01			−0.10	0.04	<0.01			−0.07	0.02	<0.01	
Age		0.01	0.01	<0.01			0.03	0.01	<0.01			0.02	0.01	<0.01	
Sex		0.03	0.05	0.63			0.02	0.05	0.66			0.02	0.03	0.45	
AgeXSex		−0.02	0.02	0.30			−0.03	0.02	0.06			−0.02	0.01	0.07	
t_0_-BMI-SDS		0.01	0.05	0.87			0.01	0.04	0.83			0.01	0.03	0.79	
COVID-19 pandemic		0.10	0.07	0.18			0.24	0.09	<0.01			0.11	0.04	<0.01	
SDQ Total		0.00	0.01	0.39			0.01	0.01	0.33			0.00	0.00	0.22	
KINDL-R Physical		0.00	0.00	0.30			0.00	0.00	0.05			0.00	0.00	0.06	
KINDL-R Mental		0.00	0.00	0.94			0.00	0.00	0.77			0.00	0.00	0.96	
HBSC-SCL		0.00	0.01	0.91			0.00	0.01	0.84			0.00	0.00	0.92	
ChEDE-Q8		−0.04	0.02	0.08			−0.06	0.02	0.01			−0.04	0.01	<0.01	
Binge Eating		0.00	0.01	0.70			0.00	0.01	0.55			0.00	0.00	0.45	
Motivation		0.01	0.01	0.32			0.02	0.01	0.20			0.01	0.01	0.15	

Notes: Intent-to-treat sample with multiple imputation; BMI-SDS = Body Mass Index-Standard Deviation Score; BOCF = Baseline Observation Carried Forward; SDQ = Strengths and Difficulties Questionnaire; KINDL-R = Health-Related Quality of Life in Children and Adolescents; HBSC-SCL = Health Behavior in School Children–Symptom Checklist; ChEDE-Q8 = Child Eating Disorder Examination-Questionnaire8.

**Table 3:** Exploratory post-hoc analysis: Correlations between ChEDE-Q8 subscales and treatment success (Δ-BMI-SDS).

	Intent-to-Treat (*n* = 361)		Completer (*n* = 214)		BOCF (*n* = 361)	
	***r***	***p***	***r***	***p***	***r***	***p***
Restrained Eating	−0.01	0.94	−0.04	0.56	−0.02	0.70
Eating Concern	0.02	0.79	−0.01	0.90	0.01	0.87
Shape Concern	0.01	0.86	−0.03	0.65	−0.02	0.76
Weight Concern	0.03	0.69	0.00	0.96	0.01	0.83

Notes: Intent-to-Treat sample with multiple imputation. ChEDE-Q8 = Child Eating Disorder Examination-Questionnaire8; BMI-SDS = Body Mass Index-Standard Deviation Score; BOCF = Baseline Observation Carried Forward.

**Table 4:** Logistic regression analysis for the predictors of treatment adherence (dropout/completer) (*n* = 361).

	*B*	*SE B*	Wald	*p*	*OR*
Model 1					
Constant	0.43	0.17	6.29	0.01	1.54
Age	0.00	0.05	0.01	0.94	1.00
Sex	0.08	0.24	0.12	0.73	1.09
AgeXSex	−0.06	0.08	0.53	0.47	0.95
t_0_-BMI-SDS	0.01	0.22	0.00	0.96	1.01
COVID-19 pandemic	−0.93	0.36	6.78	0.01	0.40
Model 2					
Constant	0.47	0.18	6.95	0.01	1.60
Age	0.02	0.06	0.15	0.70	1.02
Sex	0.01	0.26	0.00	0.98	1.01
AgeXSex	−0.06	0.08	0.58	0.45	0.94
t_0_-BMI-SDS	0.04	0.22	0.03	0.87	1.04
COVID-19 pandemic	−0.94	0.36	6.76	0.01	0.39
SDQ Total	0.01	0.03	0.07	0.79	1.01
KINDL-R Physical	0.00	0.01	0.17	0.68	1.00
KINDL-R Mental	0.00	0.01	0.07	0.80	1.00
HBSC-SCL	0.02	0.03	0.43	0.51	1.02
ChEDE-Q8	−0.13	0.11	1.36	0.24	0.88
Binge Eating	0.02	0.03	0.50	0.48	1.02
Motivation	0.00	0.06	0.00	0.95	1.00

Notes: BMI-SDS = Body Mass Index-Standard Deviation Score; SDQ = Strengths and Difficulties Questionnaire; KINDL-R = Health-Related Quality of Life in Children and Adolescents; HBSC-SCL = Health Behavior in School Children–Symptom Checklist; ChEDE-Q8 = Child Eating Disorder Examination-Questionnaire8.

## Data Availability

Due to patient data protection, research data are not shared.
